# White Matter Metabolite Ratios Predict Cognitive Outcome in Pediatric Traumatic Brain Injury

**DOI:** 10.3390/metabo13070778

**Published:** 2023-06-22

**Authors:** Luke Berger, Barbara Holshouser, Joy G. Nichols, Jamie Pivonka-Jones, Stephen Ashwal, Brenda Bartnik-Olson

**Affiliations:** 1 School of Medicine, Loma Linda University, Loma Linda, CA 92354, USA; lberger@students.llu.edu; 2 Department of Radiology, Loma Linda University Health, Loma Linda, CA 92354, USA; barbholshouser@gmail.com; 3 Department of Pediatrics, Loma Linda University Health, Loma Linda, CA 92354, USA; jgnichols@llu.edu (J.G.N.); jpivonka@llu.edu (J.P.-J.); sashwal@llu.edu (S.A.); 4 Division of Child Neurology, Loma Linda University Health, Loma Linda, CA 92354, USA

**Keywords:** magnetic resonance spectroscopy, pediatric, traumatic brain injury, global linear regression, tissue segmentation

## Abstract

The prognostic ability of global white matter and gray matter metabolite ratios following pediatric traumatic brain injury (TBI) and their relationship to 12-month neuropsychological assessments of intelligence quotient (IQ), attention, and memory is presented. Three-dimensional proton magnetic resonance spectroscopic imaging (MRSI) in pediatric subjects with complicated mild (cMild), moderate, and severe TBI was acquired acutely (6–18 days) and 12 months post-injury and compared to age-matched typically developing adolescents. A global linear regression model, co-registering MRSI metabolite maps with 3D high-resolution magnetic resonance images, was used to identify longitudinal white matter and gray matter metabolite ratio changes. Acutely, gray matter NAA/Cr, white matter NAA/Cr, and white matter NAA/Cho ratios were significantly lower in TBI groups compared to controls. Gray matter NAA/Cho was reduced only in the severe TBI group. At 12 months, all metabolite ratios normalized to control levels in each of the TBI groups. Acute gray matter and white matter NAA ratios were significantly correlated to 12-month assessments of IQ, attention, and memory. These findings suggest that whole brain gray matter and white matter metabolite ratios reflect longitudinal changes in neuronal metabolism following TBI, which can be used to predict neuropsychological outcomes in pediatric subjects.

## 1. Introduction

One of the major pathophysiologic mechanisms of injury in TBI hinges on mechanical shearing following rapid acceleration and deceleration, resulting in diffuse axonal injury (DAI) to the cytoskeleton with subsequent ionic imbalances, mitochondrial dysfunction, and functional metabolic deficits [1,2,3,4]. In the setting of mild TBI, computed tomography, along with conventional magnetic resonance imaging (MRI), may not adequately depict brain injury [5]. MRI-occult TBI damage may be evaluated using various methods. While diffusion tensor imaging allows for the evaluation of microstructural changes, proton magnetic resonance spectroscopy (H^1^ MRS) is well suited to effectively evaluate functional deficits by quantifying metabolic surrogate markers [6,7]. These include the following: N-acetylaspartate (NAA), an amino acid synthesized primarily in neuronal mitochondria which serves as a marker for mitochondrial metabolism; total creatine (phosphocreatine (PCr) and its precursor free Cr), a marker for intact brain metabolism; and total Choline (Cho), a marker for membrane synthesis and repair, inflammation, or demyelination [7].

Total creatine historically has been used as an internal standard for ^1^H MRS, as creatine and phosphocreatine are in equilibrium with ATP stores and are thought to remain constant regardless of fluctuating cellular energy status [8]. However, total creatine has been demonstrated to vary both with age and pathology, including TBI [8,9,10]. Choline levels have been shown to increase in the setting of cell turnover, acting as a marker for membrane synthesis and repair, inflammation, or demyelination [11]. Following TBI, elevated choline has been associated with traumatic or diffuse axonal injury, however, findings of elevated choline are inconsistent in the pediatric population [12,13]. 

NAA is an amino acid synthesized primarily in neuronal mitochondria, found in significant quantities throughout the central nervous system, and responsible for a wide range of functions [14,15]. The synthesis of NAA relies on the availability of acetyl-coenzyme A and adenosine triphosphate stores (ATP), and thus the concentration of NAA is reported to reflect mitochondrial function [16]. Further, NAA is involved in myelin lipid synthesis and is particularly important in the early development of the CNS during times of active myelination during childhood and adolescence [17,18]. Reduced NAA levels are typically interpreted as neuronal or metabolic dysfunction and are notably decreased in several conditions including dementia, epilepsy, and TBI [19]. However, the disruption of myelination could result in greater severity of symptoms and worsened outcomes in the pediatric population [20]. Because NAA decreases on a gradient according to the severity of injury [21], several studies have used single-voxel MRS NAA measurements as a proxy to evaluate and demonstrate a relationship between clinical variables and cognitive assessments [22,23,24]. Studies have shown that there are distinctions in the response to injury in the pediatric brain compared to the adult brain following TBI. In the pediatric population, prior studies have shown reduced NAA-based ratios in frontal white matter, corpus callosum, and various lobar regions that correlate to outcome [25,26,27]. While metabolite ratios have been useful in diagnostically and prognostically evaluating TBI, longitudinal studies using early MRSI as a prognostic tool following moderate-severe pediatric TBI have not been as extensively researched [28,29]. However, Holshouser et al. demonstrated that low NAA values in the basal ganglia measured early (<30 days) post-injury were predictive of long-term neuropsychological outcomes [24]. Children however do have significant improvements in functional status following rehabilitation, demonstrating the importance of identifying and diagnosing TBI early [30]. These studies support the evaluation of NAA and other relevant metabolite changes following pediatric TBI.

Historically, when using ^1^H MRS, one challenge is determining which region(s) should be sampled to yield the most sensitive and specific data to best determine prognosis [28]. In addition, tissue variations in voxel composition have diminished the statistical power to detect metabolite changes, exacerbated by volume of interest (VOI) misregistration in longitudinal studies. It is possible to decrease the voxel size to reduce partial volume effects, but this inextricably is linked to lower signal-to-noise ratios (SNR). By combining magnetic resonance spectroscopic imaging (MRSI) metabolite quantification with the anatomic-high spatial resolution MRI that accompanies it, and producing overlaid segmented white matter (WM), gray matter (GM), and cerebral spinal fluid (CSF) masks on the MRSI grid, these effects can be reduced [31]. Further, this information can also be processed to yield global WM and GM metabolite concentrations. In the setting of a diffuse injury such as TBI, averaging the voxels for the sake of improving SNR and partial volume effects is a reasonable tradeoff [32]. Furthermore, it has been shown recently that decreases in WM NAA TBI subjects were more sensitive to detection by global linear regression when compared to regional voxel averaging [33].

The present study investigated the accuracy of using global WM and GM metabolite ratios in a pediatric TBI subject population. This approach uses linear regression to account for voxel tissue composition and decreasing partial volume effects to calculate metabolite levels and ratios that reflect global metabolite information, eliminating the bias introduced by choosing specific anatomical regions. Analyses were performed using previously reported data [24] with the aim of evaluating the ability of global WM and GM metabolite ratios to predict 12-month neuropsychological (NP) outcomes. Given the increased sensitivity for global linear regression to detect changes in metabolites [33], we hypothesized that group differences in GM and WM metabolite ratios would be observed and would correlate to NP outcomes in TBI subjects. Moreover, we hypothesized that global WM and GM ratios would have improved predictive accuracy compared to regional analysis of MRSI.

## 2. Materials and Methods

### 2.1. Study Design

**Figure 1:**
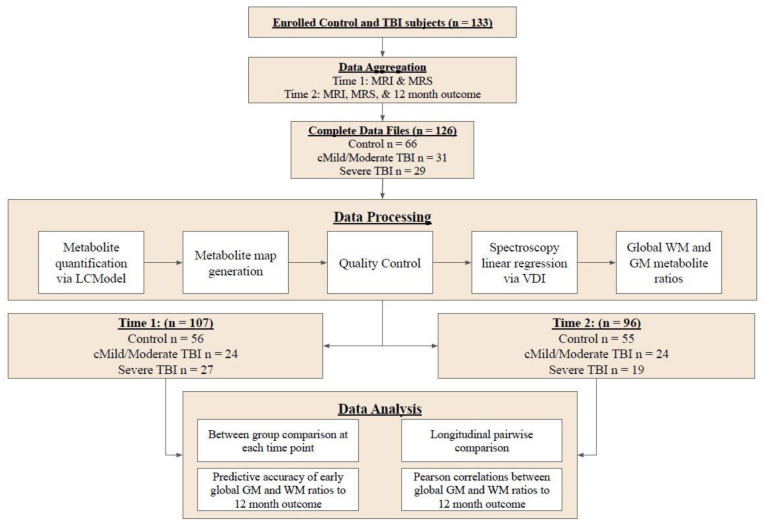
Schematic of the overall study design. GM, gray matter; MRI, magnetic resonance imaging; MRS, magnetic resonance spectroscopy; TBI, traumatic brain injury; VDI, visual display interface; WM, white matter.

Figure 1 illustrates the overall study design including data processing and data analysis. Details at each step are described in the methods below.

### 2.2. Study Population

The study data were obtained from a longitudinal, prospective study investigating metabolite changes after pediatric TBI [24]. This study recruited from hospital admissions and the surrounding community pediatric clinics in the case of healthy controls between 2010–2015. This study initially included data from 133 enrolled children (69 controls, 33 cMild/Moderate TBI, 31 Severe TBI). Seven MRSI studies were excluded due to missing and/or incomplete files; 3 from the control group, 2 from the cMild/mod TBI group, and 2 from the severe TBI group. The remaining population of 126 subjects included 29 subjects with severe TBI, 31 subjects with cMild/moderate TBI, and 66 control subjects who underwent data processing (study design Figure 1). Following spectral quality analysis, acute data from 107 subjects (severe TBI n = 27, cMild/moderate TBI n = 24, and control n = 56) were included in the subsequent time 1 data analysis. At time 2 (12-month follow-up), 96 subjects (severe TBI n = 19, cMild/moderate TBI n = 24, and control n = 55) were included in the time 2 data analysis. Only 77 subjects (severe TBI n = 17, cMild/moderate TBI n = 15, and control n = 45) completed MRSI studies at both time 1 and time 2 and were included in the longitudinal pairwise comparisons.

Inclusion criteria for TBI subjects: (1) aged between 4–18 years at the time of injury, (2) absence of previous brain injury, neurological disorder, drug or alcohol abuse, or MRI contraindications, (3) Glasgow Coma Scale (GCS) scores between 3 and 8 for severe TBI, 9 and 12 for moderate TBI, and 13–15 if hemorrhage was detected on acute CT imaging for complicated mild (cMild). The inclusion criteria for healthy, typically developing control subjects were included if they met criteria 1 and 2 and were scanned without sedation. The study was reviewed by the local institutional review board with participants providing written informed consent (if age > 11 years) or assent if aged 7–11 years, with parents providing written informed consent [24].

### 2.3. MR Acquisition

**Figure 2:**
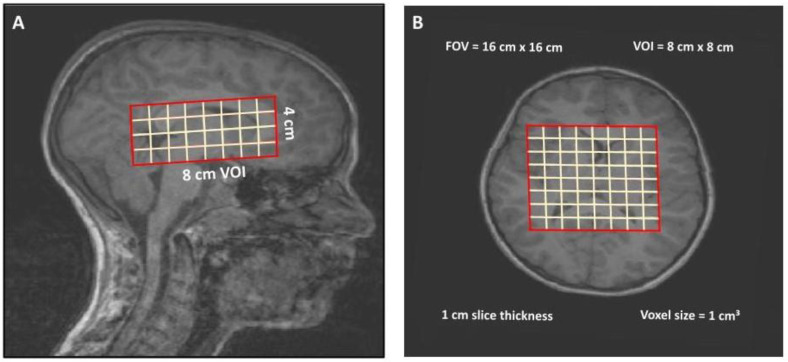
Positioning of the 3D MRSI Volume of Interest (VOI) position superimposed on T1-weighted (**A**) sagittal and (**B**) axial images. The 8 × 8 × 4 cm^3^ VOI was positioned to include the genu, body, and splenium of the corpus callosum inferiorly through the superior brainstem and including portions of the gray and white matter in the deep gray basal ganglia, thalamus, internal capsule, and periventricular white matter. Slice thickness (1 cm), nominal voxel size (1 cm^3^), and positioning were consistent for all subjects.

MR studies were performed at 3T using a 12-channel receive-only head array coil (Siemens Tim Trio; Siemens Medical Solutions, Erlangen, Germany). MRI/MRS scans were acquired on TBI subjects acutely between 6–18 days post-injury (time 1) and repeated at 1 year post-injury (time 2). Control subjects were scanned using the identical protocol and received scans at enrollment (time 1) and after a 1-year time interval (time 2). For the purposes of this study, an isotropic 3D sagittal T1-weighted inversion prepared fast spoiled gradient echo sequence (MPRAGE, TR/TE = 1950/2.26 ms, NEX = 1, voxel size 1.0 × 1.0 × 1.0 mm^3^) and 3D proton MRSI (PRESS TR = 1700 ms, TE = 144 ms, NEX = 1, 1024 data points, dwell time = 1ms) were analyzed. The MRSI VOI covered an 8 cm anterior-posterior × 8 cm left-right cm × 4 cm superior-inferior volume which was positioned over the genu, body, and splenium of the corpus callosum inferiorly through the superior brainstem, while also including portions of the gray and white matter in the deep gray basal ganglia, thalamus, internal capsule, and periventricular white matter (Figure 2). Four 1 cm thick slabs were partitioned over a 16 × 16 cm field of view (FOV) to yield individual nominal voxel sizes of 1 × 1 × 1 cm^3^, yielding a total of 128 voxels per subject (Figure 2).

### 2.4. Metabolite Quantification

Relative concentrations of NAA, total Cr, and total Cho and a percent standard deviation (%SD) were obtained for each individual voxel using LCmodel (Stephen Provencher Inc., Oakville, Ontario, Canada; LCModel Version 6.0). MRS quality checks were based on the inclusion of metabolite data with NAA %SD less than 20%. Metabolite ratios were then calculated (NAA/Cr, NAA/Cho, Cho/Cr) for each retained voxel and recorded in a spreadsheet for further analysis. Absolute quantification of each metabolite was not possible as a non-water-suppressed reference scan was not acquired in the original study. As such, metabolite ratios, which are a robust form of spectroscopic analysis [29] and not subject to effects or absence of water-scaling, were used. Voxels that failed quality control were manually counted and only subjects with at least 80% of voxels within the VOI meeting the %SD threshold were included in the analysis.

### 2.5. Generation of Metabolite Maps

A custom MATLAB (MATLAB 2021b; The MathWorks Inc., Natick, MA, USA) script using Visual Display Interface (VDI, version 1.1.3) was used to generate NAA/Cr, NAA/Cho, and Cho/Cr metabolite ratio maps overlaid on the original T1 MPRAGE image [34]. Metabolite maps were inspected for anatomical misregistration and subsequently used in the tissue linear regression (below) to generate global WM and GM metabolite ratios. At this point, the entire field of view (FOV) had been reconstructed and incorporated into the metabolite map, requiring further processing to define the proper VOI as outlined above. This was accomplished in MRIcroGL.exe (1.2.2, www.nitrc.org, accessed on 1 April 2023).

### 2.6. Spectroscopy Linear Regression

**Figure 3:**
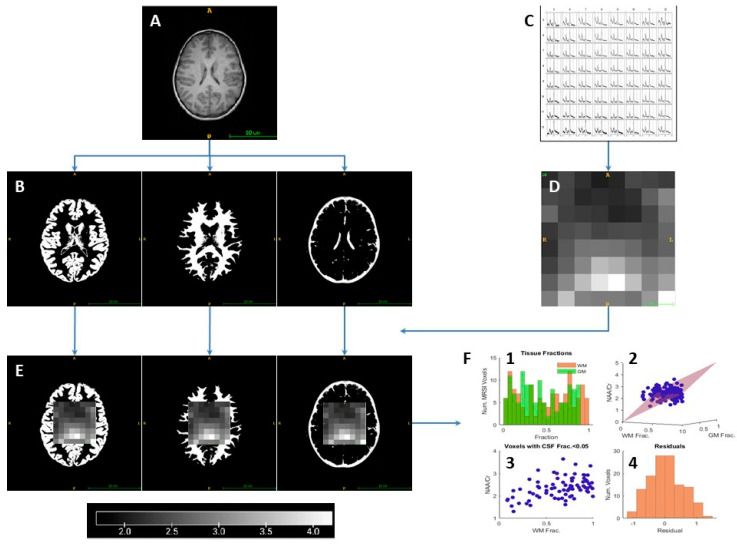
Data processing scheme. Axial T1-weighted MR images (**A**) were segmented to produce GM, WM, and CSF tissue masks (**B**). Proton MR multi-voxel MR spectroscopic imaging data was post-processed in LCmodel (**C**) and used to generate metabolite ratio maps of NAA/Cr, NAA/Cho, and Cho/Cr ratios, with pixel signal intensity used as a proxy for increased value (**D**). Metabolite maps were aligned to the T1-weighted MR imaging space and co-registered to the respective tissue masks to estimate the tissue concentration of each MRSI voxel (**E**). Using a linear regression model, global values for metabolite ratios were generated (**F**) along with a plot of the tissue fractions within each voxel (F1), the metabolite ratios versus WM, GM, and CSF (F2), the metabolite ratios versus voxels with less than 0.05% CSF (F3), and a residual plot to visualize distribution of data (F4). Cr, creatine; CSF, cerebrospinal fluid; GM, gray matter; MR, magnetic resonance; NAA, N-acetylaspartate; WM, white matter.

Key data processing steps are depicted in Figure 3 and described in greater detail below. Segmentation was performed on the T1 MPRAGE images using the Statistical Parametric Mapping open-source software (SPM2) within MATLAB to generate CSF, WM, and GM segmentation masks (Figure 3B). The metabolite ratio maps and tissue segmentation masks were aligned with the MRSI grid using the *ImageAnalysis_TissueLinearRegression* VDI MATLAB script, which counted the number of pixels for each segmented tissue mask falling into each spectroscopy voxel in the VOI to estimate tissue volume (16; Figure 3C–E). The algorithm returns extracted values for average global WM and GM metabolite ratios, accounting for spectroscopic data and underlying tissue percentages for each voxel within the VOI. An output that visualized the data graphically was used to ensure the normality of the data to be used for further analysis (Figure 3F1–F4). A more detailed explanation of this software can be found in Tal et al. [31].

### 2.7. Clinical and Neuropsychological Outcome Assessments

Clinical data recorded during the hospital admission of TBI subjects included the following: age at initial study, sex, time to MRI after injury, time to MRI follow up, average GCS, accident type, days in coma, days on ventilator, days in hospital, presence of seizures, and loss of consciousness (Table 1). Analysis of outcome by group (Table 1) was determined by twelve-month assessments of attention (Sky Search subtests from the Teach of Everyday Attention for Children [TEA-Ch-G; 6–16 years], [TEA-Ch-C; 6–16 years]), intelligence (Full-Scale Intelligence Quotient [FSIQ], Verbal Intelligence Quotient [VIQ], Performance Intelligence Quotient [PIQ]), and memory (Children’s Memory scale [5–16 years] or Wechsler Memory Scale [17–18 years]), and Pediatric Cerebral Performance Category Scale (PCPCS) scores. Memory scores were converted to z-scores to allow for statistical comparison across different measures and reported as a Combined Memory Z-Score (CMZ).

### 2.8. Quality Control and Statistical Analysis

It is well noted that missing spectroscopic data is a common finding in chemical shift imaging (CSI) [35]. While models using single-voxel or regional voxel analysis may allow for a certain degree of missing data during acquisition, the global regression VDI software used for analysis is unable to exclude single-voxel data within the defined VOI. After initial processing of the data, we recognized most subjects had compromised or missing data in the most inferior and superior slices. We suspect this was heavily influenced by underlying chemical shift artifacts. To retain subjects and homogeneity for analysis, we focused on data from the central slabs of the VOI.

SPSS (version 22; SPSS; Inc., Chicago, IL, USA) and GraphPad Prism (GraphPad Prism 9.1.0 for Windows, GraphPad Software, San Diego, California, USA) were used for all computations, while GraphPad Prism 9 was used for graphical displays. One-way analysis of covariance (ANCOVA) was used to compare the means of each metabolite ratio between controls, cMild/Moderate TBI subjects (GCS = 9–15), and severe TBI subjects (GCS = 3–8) both acutely and at 12 months, respectively. Group was used as the classification factor; metabolite ratios were selected as the dependent variable. Covariates included age, sex, and time between injury and first MRI. The assumption of homogeneity of variance was evaluated using Levene’s test to check for equal variance among metabolite ratios in respective groups. A significant result and thus rejection of the assumption of homogeneity led to analysis with Kruskal–Wallis and subsequent Mann–Whitney U tests, however, it should be noted that these tests do not factor in covariates and thus age, sex, and time between injury and first MRI were not accounted for. Non-significant results which satisfied ANCOVA assumptions were followed by Bonferroni post hoc analysis to control for multiple comparisons.

Paired sample T-tests were used to evaluate for differences within groups at the acute stage (time 1) versus the 12 months follow-up (time 2) stage. Pearson correlations were used to identify relationships between acute (time 1) metabolite ratios and 12-month clinical and NP outcomes. Given the number of individual correlations performed, initial Pearson correlation *p*-values were adjusted using a False Discovery Rate (Q = 10%) and reported as q values.

To evaluate the predictive accuracy of acute metabolite ratios, 12-month NP outcomes which had been previously dichotomized were used [24]. The dichotomized groupings were generated utilizing a 1.50 standard deviation (SD) cutoff below the normative mean, yielding scores above the cutoff (score > −1.5 SDs; not impaired) and scores below the cutoff (score < −1.49 SDs; impaired). PCPCS was dichotomized into not impaired (PCPCS = 1–3) or impaired (4–6). Utilizing acute global metabolite ratios as independent variables and dichotomized values of NP outcome as dependent variables, binary logistic regression was performed to evaluate how well the independent variables could accurately predict impaired or not impaired status.

## 3. Results

### 3.1. Clinical Demographics

Demographic information, clinical data, and 12-month NP outcomes are outlined in Table 1. The number of days in a coma, on a ventilator, and in the hospital were significantly longer in the severe TBI subjects compared to cMild/moderate (Table 1). Neurological and NP outcomes at 12 months were significantly different in severe TBI subjects compared to cMild/moderate in all measures: PCPCS (*p* < 0.001); IQ (FSIQ, PIQ, VIQ; *p* < 0.001); attention (TEA-Ch-G and TEA-Ch-C, *p* = 0.010 and *p* = 0.002; respectively) and memory (combined memory Z-score; *p* < 0.001; Table 1). Additionally, there were significantly fewer females in both the cMild/moderate and severe TBI groups when compared to the control group (Table 1) as previously reported in the literature [36].

### 3.2. Magnetic Resonance Spectroscopic Group and Longitudinal Analysis

**Figure 4:**
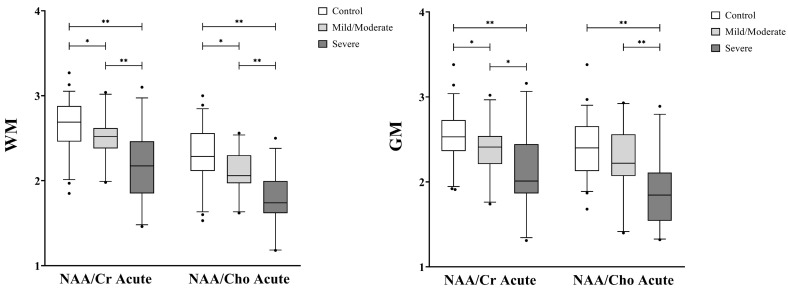
Acute global white matter (WM, **left**) and gray matter (GM, **right**) metabolite ratios in control versus complicated mild/moderate and severe TBI subjects. The box represents the 25th percentile, median, and 75th percentile, the whiskers extend from 5% to 95% of data, and the dots represent outliers. WM NAA/Cr and NAA/Cho are significantly reduced in both TBI groups compared to controls. GM NAA/Cr was significantly reduced in both TBI groups compared to controls whereas GM NAA/Cho was significantly reduced in severe TBI compared to controls or cMild/moderate TBI. Significant differences in means as determined by ANCOVA covering for age with post hoc with Bonferroni analysis, * *p* ≤ 0.02 and ** *p* ≤ 0.001. ANCOVA, analysis of covariance; cMild, complicated mild; Cr, creatine; Cho, choline, GM, gray matter; NAA, N-acetylaspartate; TBI, traumatic brain injury; WM, white matter.

**Figure 5:**
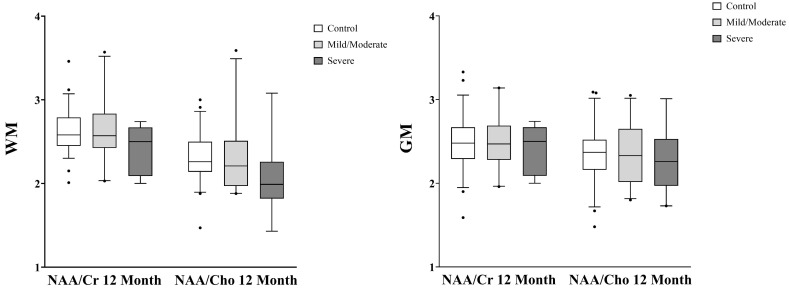
Twelve-month global white matter (WM, **left**) and gray matter (GM, **right**) metabolite ratios in control versus complicated mild/moderate and severe TBI subjects. The box represents the 25th percentile, median, and 75th percentile, the whiskers extend from 5% to 95% of data, and the dots represent outliers. There were no differences in global WM or GM NAA/Cr and NAA/Cho ratios between the control and TBI groups. Mean differences were determined by ANCOVA followed by post hoc Bonferroni analysis. ANCOVA, analysis of covariance; cMild, complicated mild; Cr, creatine; Cho, choline, GM, gray matter; NAA, N-acetylaspartate; TBI, traumatic brain injury; WM, white matter.

Figure 4 and Figure 5 display comparisons of global metabolite ratios measured within the VOI for each group. Acute WM NAA/Cr (cMild/Mod: *p* = 0.017; Severe: *p* < 0.000, Figure 4), WM NAA/Cho (cMild/Mod: *p* = 0.025; Severe: *p* < 0.000, Figure 4), and GM NAA/Cr (cMild/Mod: *p* = 0.281; Severe: *p* < 0.000, Figure 4) were significantly reduced in both TBI groups compared to controls and each other, while acute GM NAA/Cho only reached a significant reduction in the severe TBI group (*p* < 0.000, Figure 4). At the 12-month time point, the WM NAA/Cr (cMild/Mod: *p* = 1.000; Severe: *p* = 0.433, Figure 5), GM NAA/Cr (cMild/Mod: *p* = 1.000; Severe: *p* = 0.800. Figure 5) and WM NAA/Cho (cMild/Mod: *p* = 1.000; Severe: *p* = 0.194, Figure 5), and GM NAA/Cho (cMild/Mod: *p* = 1.000; Severe: *p* = 1.000, Figure 5) metabolite ratios increased to within two standard deviations of the control levels in both TBI groups, with the severe TBI group reporting the lowest metabolite ratios.

Longitudinal comparisons within group populations are displayed in Table 2. To adjust for multiple comparisons, *p*-values < 0.001 were considered to be significant. While the GM and WM NAA ratios for both TBI groups showed an increase at time 2, only WM NAA/Cr in severe TBI met the requirements for significance. It should be noted that the sample size for paired analysis was notably smaller than for group analysis, as previously described in the study population section, due to data at both time points being intrinsically required for each subject (n = 45, 15, 17 for controls, cMild/Moderate, and Severe respectively)]. Importantly, there was no statistically significant difference in control group values at the two time points, indicating that age-related changes in metabolite ratios were not a considerable factor.

### 3.3. Correlation of Metabolite Ratios to Clinical Variables and NP Outcomes

Pearson correlation (r) and adjusted *p*-values (q-values) between acute metabolite ratios and clinical variables or NP outcomes are listed in Table 3. Strong significant (*p* < 0.001) correlations (Pearson: 0.5 < r < 0.7) were observed between early global NAA ratios and numerous clinical variables (days in coma, days on ventilator, days in hospital). Global NAA ratios and NP outcomes (CMZ, FSIQ, PIQ, VIQ, TEA-Ch-G, TEA-Ch-C) were significant (*p* < 0.001), however, they were only moderately correlated (Pearson: 0.3 < r < 0.5) or even weakly correlated (Pearson: 0.1 < r < 0.3). The Cho/Cr ratios were not correlated with any of the assessments.

### 3.4. Predictive Accuracy of Metabolite Ratios on NP Outcomes

The predictive accuracy of acute metabolite ratios on 12-month NP outcomes was evaluated and reported in Table 4. In general, the specificity values were high (89.2–100%) while the sensitivity values were low (0–61.5%). The overall percent accuracy values varied between 72.5 and 92.2% with WM NAA/Cr having the highest overall percent accuracy with the highest variance explained for the majority of NP outcomes (CMZ, FSIQ, PCPCS, PIQ2, Tea-Ch-G) compared to other acute metabolite ratios.

## 4. Discussion

At present, there are several challenges impeding the path to the widespread clinical use of spectroscopy data in evaluating TBI: partial volume effects and the need for tissue segmentation [6], misregistration of a determined VOI [31], deciding which brain regions to sample when evaluating diffuse, non-focal pathologies [24], and the inherent lack of statistical power which accompanies the latter for TBI. Using a 3D multi-voxel global linear regression approach provides a mechanism to address these issues, by averaging metabolite levels over the entire VOI, properly co-registering MRSI quantification with its accompanying high-spatial-resolution MRI, and accounting for underlying tissue composition through the use of segmentation. This approach is well suited to the study of TBI as there is increasing evidence that metabolite changes following TBI are diffuse in nature [6,24,28,37]. A global linear regression approach has been previously applied in adult mild TBI [6,32,38]. To our knowledge, this is the first study to apply a linear regression approach to examine global WM and GM metabolite changes following pediatric TBI and, using this approach, we identified robust differences in acute WM and GM NAA ratios between all groups with an exception between control and cMild/Moderate TBI for GM NAA/Cho. Given that Cho increases in the setting of axonal damage [11], NAA/Cho is typically a sensitive measure of diffuse axonal injury, and thus it is possible that cMild/Moderate TBI subjects experienced less damage in GM than WM.

It has been suggested that there is a threshold of injury beyond which cells are unable to functionally recover and restoration of metabolite levels is not possible [39]. In the present study, we found that after 12 months post-injury there were no longer significant differences in global WM or GM NAA/Cr and NAA/Cho between controls and either TBI group, suggesting some recovery of neuronal function. However, despite increases in mean metabolite ratios, significant group differences in neurocognitive outcome were present at 12 months with severely injured subjects being most affected. Prior studies have suggested that similar discrepancies between metabolite findings and outcome measures may represent ongoing, alternative pathophysiological mechanisms of TBI injury which are obscured from metabolite detection but related to the myriad of secondary injuries including vascular permeability, osmolarity changes, intracranial pressure differences, excitotoxicity, oxidative stress, and inflammation [40].

We also examined the relationship between acute global WM and GM NAA-associated ratios to 12-month NP outcomes as well as the predictive accuracy of the global ratios. While all NAA ratios were significantly correlated to several NP measures, global WM NAA/Cr ratios showed significant moderate or strong correlations with all clinical variables and 12-month NP outcomes. Additionally, global WM NAA/Cr ratios appear to be useful in terms of predictive accuracy for long-term NP outcomes, as demonstrated by consistently high overall percent accuracy and specificity. When compared to the results from traditional 3D-regional MRSI, the current study yielded values of predictive accuracy that are similar, thus providing clinical utility more effective at predicting recovery than long-term impairment [24]. Compared to the specificity and overall accuracy, sensitivity values were much lower. We hypothesize this is related to a smaller difference in means between control and cMild/Moderate TBI subjects in contrast to the larger difference between control and severe TBI subjects. Despite the overall significance of the model, it is plausible that the logistic regression classified cMild/Moderate TBI subjects into the control category, and thus discrimination was difficult [41]. As sample size influences logistic regression accuracy to reflect target population parameters, misclassification between these groups may also partially reflect the study populations which consisted of more control (n = 66) compared to cMild/Moderate TBI subjects (n = 31) [42]. In the absence of severe injury, it would suggest that the global linear regression model has less prognostic certainty for evaluating mild/moderate TBI. However, the relative success of global WM NAA/Cr in both correlation and predictive accuracy is consistent with previous studies, suggesting that shearing forces that lead to diffuse axonal injury may reflect WM’s increased vulnerability to disruption and correlation to long-term neurocognitive outcomes. As a result, global measures of WM NAA/Cr are especially likely to be helpful, especially in the setting of severe TBI.

The limitations of the study include its relatively small sample sizes, especially in the longitudinal analysis, which did not allow us to form more conclusive opinions on our findings. The small sample size was due, in part, to missing metabolite data and the removal of subjects if <80% of the voxels did not meet the quality control limits. Initially, we suspected regions without metabolite data were the result of a focal injury such as hematoma or hemorrhage, since spectroscopy cannot reliably sample these regions [43]. However, we also noted that there was an increase in the number of excluded data sets at 12 months for TBI subjects, and it is possible that these subjects may have had difficulty remaining motionless during the MRSI acquisition, leading to poor quality spectra. Finally, age at injury was not factored into binary logistic regression and does represent a possible confounding variable. Developmental plasticity represents adaptive structural and functional changes in the brain in response to internal or external stimuli [44], including injury such as TBI. Plasticity is both time- and age-dependent and it is plausible that the ability of the brain to recover following injury is influenced by age. Thus, it is likely that both metabolite ratios and NP outcomes would demonstrate variable responses based on age.

## 5. Conclusions

Our findings are based on a relatively small sample but provide compelling evidence that supports the use of global linear regression modeling of MRSI data to effectively detect acute alterations in metabolite levels following pediatric TBI. Moreover, the results support the indication that early alterations in these metabolite levels reflect neuronal and cellular integrity, rather than cell death [21,43], which can be used as a proxy for estimating NP outcomes in pediatric subjects. Although this technique prioritizes sensitivity at the expense of localization, it provides a promising framework for the efficient detection of widespread, diffuse injury which may otherwise be undetectable.

## Figures and Tables

**Table 1:** Subject demographics by group. Values are reported as means ± standard deviation. Differences in sex, presence of seizures, and loss of consciousness between groups were determined using Chi-squared analysis. Differences in age were determined using one-way ANOVA. For the remainder of the variables, differences between groups were determined using non-parametric Kruskal-Wallace or ANCOVA using age as a covariate. Significance was reached at *p* ≤ 0.05. ANCOVA, analysis of covariance; ANOVA, analysis of variance; ATV, all-terrain vehicle; FSIQ, Full-Scale Intelligence Quotient; GCS, Glasgow Coma Scale; MRI, Magnetic Resonance Imaging; MVA, motor vehicle accident; MV, motor vehicle; PCPCS, Pediatric Cerebral Performance Category Scale Score; PIQ, Performance Intelligence Quotient; TBI, Traumatic brain injury; TEA-Ch, Test of Everyday Attention for Children; VIQ, Verbal Intelligence Quotient.

	Control (n = 66)	cMild/Moderate TBI (n = 31)	Severe TBI (n = 29)	*p*-Value
Age (years) at initial study (range, median)	12.69 ± 3.34 (5.5–18.4, 13.4)	11.75 ± 3.46 (5.2–17.2, 12.2)	12.15 ± 3.49 (5.8–17.8, 12.9)	0.421
Sex	33M/33F	24M/7F	20M/9F	**0.015**
Time (days) to MRI after injury (range, median)	NA	11.45 ± 3.10 (6–17, 11)	10.90 ± 3.37 (6–18, 10)	0.509
Time (months) to follow up MRI (range, median)	12.76 ± 1.08 (11.0–16.7, 12.62)	12.06 ± 0.93 (10.7–14.5, 11.9)	12.17 ± 0.64 (10.8–13.3, 12.2)	**0.002**
Average GCS score	NA	13.77 ± 1.86	4.45 ± 1.86	**0.000**
Accident Type	NA	11 falls, 6 MVA, 6 hit by MV, 5 sports, 2 ATV, 1 fight	3 falls, 8 MVA, 15 hit by MV, 2 ATV, 1 boating	
Days in coma	NA	0.65 ± 0.55	5.28 ± 5.84	**<0.001**
Days on ventilator	NA	0.00 ± 0.0	4.90 ± 4.42	**<0.001**
Days in hospital	NA	6.00 ± 2.96	16.55 ± 9.94	**<0.001**
Seizures (epilepsy)	NA	4 (0)	8 (2)	0.1554
Loss of consciousness (none, <24 h, >24 h)	NA	(12, 18, 1)	(0,13,16)	**<0.001**
PCPCS @ 12 months	1.00 ± 0.0	1.06 ± 0.25	1.43 ± 0.50	**<0.001**
TEA-Ch-G @ 12 months	11.22 ± 3.14	11.72 ± 2.99	9.12 ± 3.94	**0.010**
TEA-Ch-C @ 12 months	10.92 ± 2.97	11.07 ± 2.99	8.15 ± 3.85	**0.002**
Combined Memory Z Score @ 12 months	1.19 ± 1.00	0.81 ± 1.08	−0.36 ± 1.61	**<0.001**
FSIQ @ 12 months	108.88 ± 15.20	96.10 ± 14.43	91.34 ± 14.54	**<0.001**
PIQ @ 12 months	108.35 ± 15.29	99.52 ± 15.72	94.48 ±15.47	**<0.001**
VIQ @ 12 months	108.12 ± 14.46	93.77 ± 15.72	90.00 ± 14.97	**<0.001**

**Table 2:** Pairwise comparisons between acute (time 1) and 12-month (time 2) global white matter (WM) and gray matter (GM) metabolite ratios in control, complicated mild/moderate, and severe TBI subjects. Severe TBI subjects showed a significant increase in WM NAA/Cr over the 12-month follow-up period. WM NAA/Cho increased significantly in both cMild/moderate and severe TBI NAA/groups over the 12-month follow-up period. GM NAA/Cho also increased significantly in the severe TBI group over the 12-month period. There were no significant differences in WM or GM Cho/Cr of either TBI group over time. Significant differences in means were determined by paired-t tests or non-parametric Wilcoxon tests (†) adjusted for multiple comparisons using a *p*-value cutoff of 0.001. cMild, complicated mild; Cr, creatine; Cho, choline, GM, gray matter; NAA, N-acetylaspartate; WM, white matter.

**NAA/Cr**				
	**Group**	**Acute**	**12 Month**	***p*-Value**
WM	Control (n = 45)	2.66 ± 0.32	2.64 ± 0.27	0.790
	cMild/Moderate (n = 15)	2.49 ± 0.34	2.66 ± 0.38	0.118
	Severe † (n = 17)	2.22 ± 0.37	2.48 ± 0.24	**0.000**
GM	Control (n = 45)	2.50 ± 0.28	2.48 ± 0.41	0.826
	cMild/Moderate (n = 15)	2.36 ± 0.36	2.57 ± 0.29	0.115
	Severe † (n = 17)	2.24 ± 0.44	2.42 ± 0.29	0.055
**NAA/Cho**				
	**Group**	**Acute**	**12 Month**	***p*-Value**
WM	Control (n = 45)	2.30 ± 0.35	2.33 ± 0.29	0.459
	cMild/Moderate (n = 15)	2.07 ± 0.30	2.26 ± 0.26	**0.040**
	Severe (n = 17)	1.83 ± 0.29	2.04 ± 0.41	**0.006**
GM	Control (n = 45)	2.39 ± 0.33	2.36 ± 0.34	0.660
	cMild/Moderate (n = 15)	2.19 ± 0.45	2.34 ± 0.28	0.284
	Severe † (n = 17)	1.97 ± 0.44	2.30 ± 0.34	**0.010**
**Cho/Cr**				
	**Group**	**Acute**	**12 Month**	***p*-Value**
WM	Control (n = 45)	1.18 ± 0.14	1.16 ± 0.11	0.298
	cMild/Moderate (n = 15)	1.24 ± 0.16	1.21 ± 0.10	0.470
	Severe (n = 17)	1.24 ± 0.16	1.25 ± 0.19	0.710
GM	Control (n = 45)	1.25 ± 0.15	1.24 ± 0.13	0.737
	cMild/Moderate (n = 15)	1.35 ± 0.22	1.33 ± 0.17	0.654
	Severe † (n = 17)	1.57 ± 0.93	1.33 ± 0.20	0.238

**Table 3:** Pearson correlations (r) between tissue-specific acute global NAA metabolite ratios, clinical variables, and 12-month neuropsychological outcomes. Global WM NAA/Cr and NAA/Cho were significantly associated with all clinical variables and 12-month neuropsychological outcome measures, except for WM NAA/Cho and TEA-Ch-G. Acute global GM NAA/Cr was significantly associated with all clinical variables and 12-month neuropsychological outcome measures, except for GCS and VIQ. Similarly, acute global GM NAA/Cho was significantly associated with most clinical variables and 12-month neuropsychological outcome measures, except for TEA-Ch-G. *p*-values were adjusted using a false-determination rate (FDR) and an alpha level set at α < 0.001 to control for the multiple comparisons made and reported as q-values. FSIQ, Full-Scale Intelligence Quotient; GCS, Glasgow Coma Scale; PCPCS, Pediatric Cerebral Performance Category Scale Score; PIQ, Performance Intelligence Quotient; TEA-Ch, Test of Everyday Attention for Children; VIQ, Verbal Intelligence Quotient.

	WM NAA/Cr		WM NAA/Cho		GM NAA/Cr		GM NAA/Cho	
	r	q	r	q	r	q	r	q
**GCS**	0.497	**<0.001**	0.535	**<0.001**	0.419	0.002	0.455	**0.001**
**Days in coma**	−0.536	**<0.001**	−0.558	**<0.001**	−0.561	**<0.001**	−0.569	**<0.001**
**Days on ventilator**	−0.533	**<0.001**	−0.583	**<0.001**	−0.578	**<0.001**	−0.624	**<0.001**
**Days in hospital**	−0.599	**<0.001**	−0.619	**<0.001**	−0.566	**<0.001**	−0.605	**<0.001**
**Combined Memory Z Score**	0.495	**<0.001**	0.345	**<0.001**	0.468	**<0.001**	0.448	**<0.001**
**FSIQ**	0.471	**<0.001**	0.387	**<0.001**	0.411	**<0.001**	0.355	**<0.001**
**PIQ**	0.399	**<0.001**	0.308	**0.001**	0.461	**<0.001**	0.363	**<0.001**
**VIQ**	0.429	**<0.001**	0.351	**<0.001**	0.289	0.004	0.278	**<0.001**
**TEA-Ch-G**	0.371	**<0.001**	0.279	0.01	0.456	**<0.001**	0.292	0.007
**TEA-Ch-C**	0.471	**<0.001**	0.369	**<0.001**	0.551	**<0.001**	0.367	**<0.001**

**Table 4:** Binary Logistic Regression to Predict 12-month Neurological and Neuropsychological Outcomes in TBI patients. All metabolite ratios were measured at the acute time point (time 1). Percent of variance explained was estimated using the Nagelberke R square. Cr, creatine; Cho, choline, GM, gray matter; NAA, N-acetylaspartate; WM, white matter. FSIQ, Full-Scale Intelligence Quotient; GCS, Glasgow Coma Scale; PCPCS, Pediatric Cerebral Performance Category Scale Score; PIQ, Performance Intelligence Quotient; TBI, Traumatic brain injury; TEA-Ch, Test of Everyday Attention for Children; VIQ, Verbal Intelligence Quotient.

12 Month Dichotomized Outcomes	Percent Sensitivity	Percent Specificity	Overall Percent Accuracy	*p* Value for Model	Percent of Variance Explained
**Combined Memory Z Score**					
NAA/Cr WM	37.5	95.1	85.7	**0.001**	32.3
NAA/Cr GM	12.5	97.6	83.7	**0.018**	18.4
NAA/Cho WM	12.5	100	85.7	**0.045**	12.4
NAA/Cho GM	12.5	100	85.7	**0.040**	14
Cho/Cr WM	0	100	83.7	0.129	7.8
Cho/Cr GM	0	100	83.7	0.650	0.7
**TEA-Ch-G Attention**					
NAA/Cr WM	20	95.1	87	**<0.001**	47.8
NAA/Cr GM	0	100	89.1	**0.030**	19.5
NAA/Cho WM	20	97.6	89.1	**0.004**	33.8
NAA/Cho GM	0	100	89.1	0.078	13.1
Cho/Cr WM	0	100	89.1	0.721	0.6
Cho/Cr GM	0	100	89.1	0.571	1.4
**FSIQ**					
NAA/Cr WM	16.7	100	90.2	**0.010**	23.8
NAA/Cr GM	0	100	88.2	**0.022**	18.9
NAA/Cho WM	0	100	88.2	**0.043**	15
NAA/Cho GM	0	100	88.2	0.062	12.8
Cho/Cr WM	0	100	88.2	0.490	1.8
Cho/Cr GM	0	100	88.2	0.624	0.9
**PCPCS**					
NAA/Cr WM	61.5	94.6	86	**<0.001**	53
NAA/Cr GM	46.2	91.9	80	**<0.001**	47.1
NAA/Cho WM	61.5	89.2	82	**<0.001**	52.6
NAA/Cho GM	53.8	89.2	80	**<0.001**	41.3
Cho/Cr WM	0	100	74	0.965	0
Cho/Cr GM	0	100	74	0.605	0.8
**VIQ2**					
NAA/Cr WM	30.8	94.7	78.4	**0.003**	23.1
NAA/Cr GM	15.4	97.4	76.5	0.054	10.3
NAA/Cho WM	7.7	97.4	74.5	0.106	7.4
NAA/Cho GM	15.4	100	78.4	**0.019**	15.1
Cho/Cr WM	7.7	94.7	72.5	**0.031**	12.8
Cho/Cr GM	7.7	100	76.5	0.271	3.5
**PIQ2**					
NAA/Cr WM	16.7	100	90.2	**0.008**	24.6
NAA/Cr GM	33.3	100	92.2	**0.012**	22.8
NAA/Cho WM	16.7	100	90.2	**0.023**	18.7
NAA/Cho GM	0	100	88.2	**0.046**	14.6
Cho/Cr WM	0	100	88.2	0.679	0.6
Cho/Cr GM	0	100	88.2	0.437	2.3

## Data Availability

The data presented in this study are available on request from the corresponding author. The data are not publicly available due to restrictions with private health information (PHI).
